# Accurate Sybil Attack Detection Based on Fine-Grained Physical Channel Information

**DOI:** 10.3390/s18030878

**Published:** 2018-03-15

**Authors:** Chundong Wang, Likun Zhu, Liangyi Gong, Zhentang Zhao, Lei Yang, Zheli Liu, Xiaochun Cheng

**Affiliations:** 1 Key Laboratory of Computer Vision and System, Ministry of Education, Tianjin University of Technology, Tianjin 300384, China; michael3769@163.com (C.W.); kurtcobian4ever@163.com (L.Z.); deviltangv@163.com (Z.Z.); guashushang89757@163.com (L.Y.); 2 Tianjin Key Laboratory of Intelligence Computing and Novel Software Technology, Ministry of Education, Tianjin University of Technology, Tianjin 300384, China; 3 College of Computer and Control Engineering, Nankai University, Tianjin 300350, China; liuzheli@nankai.edu.cn; 4 Department of Computer Science, Middlesex University, London NW4 4BT, UK; X.Cheng@mdx.ac.uk

**Keywords:** channel state information, Sybil attack, indoor AoA technology, DBSCAN algorithm

## Abstract

With the development of the Internet-of-Things (IoT), wireless network security has more and more attention paid to it. The Sybil attack is one of the famous wireless attacks that can forge wireless devices to steal information from clients. These forged devices may constantly attack target access points to crush the wireless network. In this paper, we propose a novel Sybil attack detection based on Channel State Information (CSI). This detection algorithm can tell whether the static devices are Sybil attackers by combining a self-adaptive multiple signal classification algorithm with the Received Signal Strength Indicator (RSSI). Moreover, we develop a novel tracing scheme to cluster the channel characteristics of mobile devices and detect dynamic attackers that change their channel characteristics in an error area. Finally, we experiment on mobile and commercial WiFi devices. Our algorithm can effectively distinguish the Sybil devices. The experimental results show that our Sybil attack detection system achieves high accuracy for both static and dynamic scenarios. Therefore, combining the phase and similarity of channel features, the multi-dimensional analysis of CSI can effectively detect Sybil nodes and improve the security of wireless networks.

## 1. Introduction

With the widespread deployment of the Internet-of-Things (IoT), its security issues have become increasingly prominent. Due to the broadcast nature of the IoT network, the privacy and security of clients will be severely threatened. Although the WLAN network is equipped with the 802.11i security protocol [1], there are still vulnerabilities in the agreement. Specifically, the Sybil attack is discussed in peer-to-peer systems by J.R.Douceur [2], which means that a client forges multiple wireless devices. The forged devices may be an existing device in the WLAN network or a virtual attack device. Each fake device can be used as a middleman to steal real clients’ private information, and when a large number of forged devices access the networks and send large amounts of data, this will block the communication of other devices in the wireless networks, which is also a kind of association flood Denial of Service (DoS) attack [3].

The previous Sybil attack detection algorithms are almost all based on the Received Signal Strength Indicator (RSSI) [4,5,6,7]. However, if there is no accurate location model, those algorithms only detect the attack by collecting fingerprints, and their detection range and robustness have great limitations. Meanwhile, traditional RSSI values will not be stable in a complex indoor environment when a filtering strategy technology is not applied [8]. Because of the complexity of the indoor wireless environment, the difficulty of Sybil attack detection is more serious. Fortunately, Channel State Information (CSI)-based indoor location technology has become more mature in recent years, and this technology can provide more accurate location results. At the same time, it achieves the passive physical layer positioning method without additional positioning equipment. Chronos [9] first achieved decimetre-level positioning in a single commercial WiFi device. Now, SpotFi [10] can achieve a median accuracy of 40 cm by estimating the Time of Flight (ToF) and Angle of Arrival (AoA) for WiFi devices.

As a result, we propose a novel Sybil attack detection algorithm based on CSI. The algorithm can detect attackers separately from both static and dynamic aspects. It is different from our previous attack detection method [11]. The biggest difference between the two papers lies in the wider application scenarios and the improved Sybil attack detection system. Although our previous paper can detect some static Sybil nodes in the environment by combining AoA and RSSI, they can only be applied to situations where there is no movement in the WLAN environment, that is the attack nodes will not be detected during the moment of their movements. Based on this, this paper put forward a complete Sybil attack detection system and dynamic attack detection algorithm, which can not only be applied to static attacks, but also to dynamic attacks. At the same time, the problem of RSSI instability in our previous paper is also solved by combining the amplitude in this paper, which makes the detection results more stable and can be more generally applicable to complex WLAN environments.

First of all, in this paper, we combine the CSI and RSSI values to remove the DC noise in the static channel state. By eliminating the sampling frequency deviation and sampling time deviation [12], we propose a self-adaptive Multiple Signal Classification (MUSIC) algorithm detection scheme for passive detection. The algorithm can effectively determine the angle of the user in the WLAN environment, and the implementation of the algorithm only needs to be detected by one AP. We distinguish between APs in different locations according to the difference of RSSIs and amplitudes in different locations and AoA technology [13,14]. This way, we can accurately distinguish between users and attackers in different locations. Secondly, on the dynamic side, it is well known that an attack may move its position within a detection threshold to avoid static detection. Therefore, we use the Density-Based Spatial Clustering of Applications with Noise (DBSCAN) clustering method [15] for all attack nodes constructed by Sybil clients that fall within the detection threshold and within the RSSI threshold. The clustering method distinguishes the attacking nodes from the normal nodes simultaneously. The algorithm enhances the overall detection accuracy.

At the same time, we propose a Sybil attack detection system. The system was able to passively detect WiFi attacks on users or AP, both static and dynamic, in real time. Finally, we experimentally test Sybil attacks at different positions and angles of the algorithm. Experiments show that physical layer-based detection can ignore any attacks on the protocol layer and the MAC layer. It can accurately detect dynamic and static attacks.

Our main contributions are summarized as follows.

- We propose a novel Sybil attack detection based on WLAN physical layer information. It can detect the static and dynamic attackers without any dedicated infrastructure.
- We develop a novel self-adaptive MUSIC algorithm to calculate the phase offset between the antennas. It can estimate the angle of wireless devices more accurately than traditional MUSIC algorithms.
- We realize a Sybil attack detection system on a common commercial platform. Extensive experiments prove that the performance of the Sybil detection is good.

The rest of paper will be carried out in the following sections: Related work is discussed in Section 2. Preliminary channel state information and Sybil attack models are given in Section 3. Section 4 is the Sybil attack detection system. Based on this, we propose a static attack detection algorithm in Section 5 and a dynamic attack detection algorithm in Section 6. Section 7 contains the experiment simulation. We will summarize in Section 8.

## 2. Related Work

As mentioned in the previous section, Sybil attacks [2] are first proposed on a distributed P2P system. It verifies that the Sybil attack occurs in the distributed storage algorithms without authentication, which can easily destroy the distributed key hash table of the system. Then, Karlof and Wagner [16] proved that distributed routing and geographic routing protocols can also be easily attacked by Sybil attacks in WSN. Newsome [17] contrasts multiple intermediate defence mechanisms such as radio resource testing and random key pre-distribution. Random key pre-distribution, which is authenticated by each node ID, has obtained relatively good results. However, each node needs to store and generate a large number of keys, which severely consumes the resources of the system. Meanwhile, the algorithm cannot resist the replay attack. Other algorithms based on authentication mechanisms [18,19] also have a similar defect as long as this is a reinforcement for the protocol layer. Pecori R. [20] proposes new methods of key agreement protocol, which can effectively resist Sybil attacks for P2P VoIP application from the transport layer. At the same time, another of his methods [21] makes the routing and the storage and retrieval of resources in a Kademlia network more secure through exploiting reputation techniques for the Sybil attack in the network layer. With the development of public cloud storage [22] and new schemes for outsourced databases recently, the new encryption methods [23,24,25] and a practical oblivious RAM with a variable block size [26] may solve the privacy and security problems caused by the Sybil attack. This paper mainly discusses the attack detection of the physical layer in the WLAN [27], so the application of these algorithms will not be done.

In recent years, Sybil attacks have been extensively studied in Wireless Sensor Networks (WSN), ad hoc networks, Vehicular Ad Hoc Networks(VANETs) [28] and Wireless Body Area Networks (WBANs) [29]. Most of the detection schemes are active and static in collecting user information, which may be data packets or RSSI values. RSSI has the advantages of lightweight and accurate detection when applied to attack detection. Consequently, in WSN, Demirbas et al. [4] firstly judged Sybil nodes by comparing the RSSI ratio differences between the neighbours’ nodes in the past. This method requires at least four detection nodes to achieve higher detection accuracy, and the wireless network environment must be arranged in advance. Furthermore, Shi [30] was able to detect attacks in the initialization phase when combining the Low Energy Adaptive Clustering Hierarchy (LEACH) protocol features with RSSI-ID tables. Still in WSN, Gu [31,32] used the K-nest and SVM algorithm in RSSI to achieve high detection efficiency and low error detection rate. However, the algorithm consumes too much detection time. In other methods, the ToA or TDoA detection algorithm [33,34] did not collect RSSI and used special positioning devices to collect every node’s angle information, including Sybil nodes. Obviously, the attacker will deliberately avoid being collected. In ad hoc networks, Mevlut [7] proposed an system INTERLOC for automatic adjustment of the positioning precision according to the level of environmental interference, which can effectively improve the detection precision of Sybil nodes. In VANET, RSSI-based detection methods are also widely used. Combining the statistical method with the RANSAC algorithm for RSSI, Yu, B. et al. [35] can distinguish the image of traffic congestion caused by attack detection. However, this method does not detect when the attack node is close to the physical vehicle. The work in [36] adds multiple detection nodes to collect the RSSI of the vehicle to determine whether it is consistent with the beacon node. Feng, X. et al. [37] used an event-based reputation system to defend against Sybil attacks. They can keep the vehicle’s identity private. However, these methods always need to rely on certain trust relationships, and the amount of information stored by each node is too large. Furthermore, by combining the RSSI of different transmit powers, Liu, R. et al. [38] combated Sybil attacks against stealing user privacy and health data in WBAN. Xiao, L. et al. [39] proposed a channel-based authentication technology to detect Sybil attacks in wireless networks, using the uniqueness of channel response in WLAN. Although a single identity node can only correspond to one location and the RSSI is proportional to the distance, the RSSI is not stable in the time domain and may differ at different times.

Meanwhile, there are many mobile elements [40,41] in most wireless networks. Once node movement exists in the environment, the traditional static algorithms of RSSI and other information will be invalid. Jamshidi, M. [42] introduced the mobile watch node into the WSN, which realizes a true negative detection rate of 94% and a false detection of zero for the movement of Sybil nodes.

## 3. Preliminaries

### 3.1. Channel State Information

RSSI belongs to the MAC layer information, which is easy to obtain, but the stability is low. When the environment becomes complex, it will be subject to the influence of multipath effects in the environment, and RSSI will be the superposition value of multipath signals. However, CSI is the application of physical layer OFDM with a finer granularity of 30 subcarriers. It can distinguish signals from multiple different paths. Specifically, CSI can be described as:

(1) $$Y\left(f_{\kappa }\right)=H\left(f_{\kappa }\right)X\left(f_{\kappa }\right)+N$$

$X(f_{\kappa })$ is used as the input signal; $Y(f_{\kappa })$ is the accepted signal; $H(f_{\kappa })$ is the channel state information matrix on each subcarrier; *N* is the environmental noise. Therefore, the CSI of each subcarrier can be approximated as:

(2) $$H\left(f_{\kappa }\right)\approx \frac{Y(f_{\kappa })}{X(f_{\kappa })}$$

According to the 802.11n physical layer protocol, the CSI will be divided into 56 subcarriers in the 20-MHz band mode. Each carrier frequency is $f_{k}=f_{centre}+0.3125k$, where *k* corresponds to the carrier index from −28–1, 1–28. Each carrier interval is 0.3125 MHz. $f_{centre}$ is the centre of the carrier frequency.

Intel 5300 NIC output is the CSI of 30 subcarriers because the CSI value is one output for each of the two adjacent carriers. That is, the received CSI is $H=[H\left(f_{1}\right)\cdots H\left(f_{k}\right)]$. Similarly, if the frequency band of 40 MHz is used, according to the protocol, the number of subcarriers will be 114, and the carrier interval remains unchanged. By using MIMO technology, we assume that the number of transmitting antennas and receiving antennas is $N_{tx}$ and $N_{rx}$, then the CSI dimension of the multi-antenna is $N_{tx}\times N_{rx}\times 30$. $H_{k}=∥H_{k}∥e^{-j\sin(∠H_{k})}$, $\sin(∠H_{k})$ and $∥H_{k}∥$ are the phase and amplitude of the *K* subcarrier respectively.

The phase will be what we are going to use in the MUSIC algorithm. The Channel Impulse Response (CIR) is obtained by inverse Fourier transform of the Channel Frequency Response (CFR). Then, the multipath channel in the time domain can be expressed as $h\left(\tau \right)=\sum_{i=1}^{N}a_{i}e^{-j\theta_{i}\left(\tau -\tau_{i}\right)}$, and the multipath function CIR reflects the multipath channel condition at the receiver. $a_{i}$, θ, $\tau_{i}$ are expressed as the amplitude, phase offset and transmission delay of the *i*-th path. *N* is the total number of transmission paths.

Recently, Halperin obtained the Channel State Information (CSI) by modifying the Intel 5300 network card driver from the ordinary WiFi device. In 802.11a/g/n, the CSI is the sampled version of CFR. Meanwhile, the Atheros CSI tool proposed by Yaxiong is supposed for all types of Atheros 802.11n WiFi chipsets now. Since the subcarriers of different frequencies are not exactly the same in space, it is possible to better characterize the space by CSI and improve the accuracy of WiFi localization.

### 3.2. Sybil Attack Models

**Figure 1:**
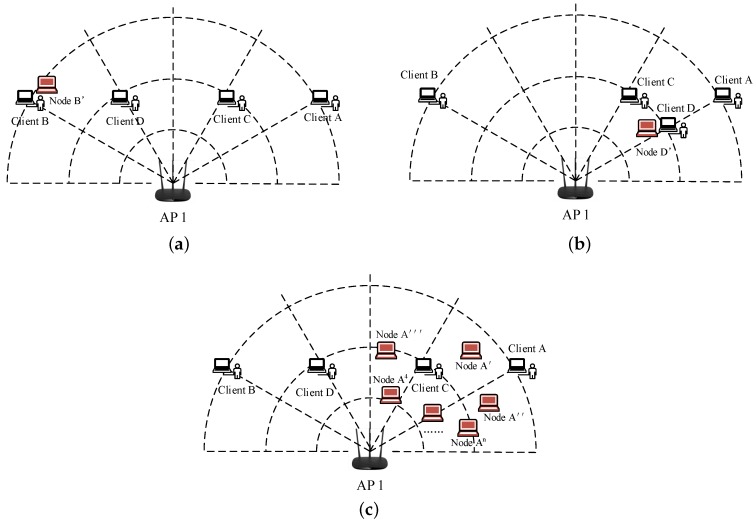
Sybil attack models. (**a**) Attack for different angles; (**b**) attack for the same angles; (**c**) a large number of Sybil nodes.

In the wireless network, most clients have a fixed position. The Sybil attacker may forge the identity of normal clients, such as IP addresses, MAC addresses or public keys. We present generalized Sybil attack models in Figure 1. We assume that there are four clients A, B, C, D in the indoor wireless network, which are located at $\left[30^{\circ },150^{\circ },60^{\circ },120^{\circ }\right]$ of the witnessing AP 1. Client A and client B are located in a 3-m radius of the circle. Similarly, client C and client D are located in a 2-m radius of the circle. AP 1 is the centre of these circles. It receives service requests from those clients in a period of time.

In Figure 1a, we assume Client A is a Sybil client, which is located $30^{\circ }$ of AP 1 and steals the identity of Client B. When Client A forged Client B’s ID as node $B^{\prime }$, Client A sent massive data to AP 1. AP 1 thinks the data are from Client B and exchanges massages with it. This is a spoofing attack, and node $B^{\prime }$ is the Sybil node. Spoofing attacks may also occur at different radians of the same angle above the radius. Unlike Figure 1a, Client A, Client D is located in the same degree $30^{\circ }$. Figure 1b shows that Client A forges the identity information of Client D and appears as node D’. Therefore, the information exchanged between Client D and AP 1 can be tapped by node D’.

As shown in Figure 1c, it is also assumed that Client A is a Sybil client. Client A claims that it has multiple identities such as node $A^{\prime }$, node $A^{\prime \prime }$, node $A^{\prime \prime \prime }$, and so on, which can consume the resources of witnessing AP 1 by sending massive high-rate service requests. Clients B, C, D will fail to access the network especially when the number of Sybil nodes is large enough. This is the typical generalized Sybil attack. Simultaneously, those nodes forged by Client A are Sybil nodes.

The attack’s Sybil nodes and part of normal clients may distribute in a small range. If Sybil nodes $A^{\prime }$, $A^{\prime \prime }$, $A^{\prime \prime \prime }$ forged by Client A gather together in Figure 1c, they are distributed in a tiny space around Client C, so we need to detect attackers from a dynamic perspective. In general, this situation happens rarely. However, when the attacker tries to move its position to change the character of the channel, the above situation still cannot be captured.

Therefore, according to the core principle of attack, the Sybil nodes forged by the attacker come from the same physical device, and they must appear or disappear simultaneously. Attack clients have the same physical layer characteristics, such as the same angle and the same distance from the AP. Therefore, whether we can accurately detect a client’s AoA will be the key to identifying Sybil attacks. As we know, in the radar system, the MUSIC algorithm [43] can detect the AoA by using the phase difference between the antennas. Therefore, the receiver uses three antennas. At the same time, we combine the MUSIC algorithm with MIMO technology to detect each user’s AoA.

### 3.3. Angle of Arrival Measurement

The main idea of the MUSIC algorithm is to calculate the covariance matrix of the received signal to separate the signal subspace and the noise subspace. Then, the orthogonality of the two subspaces is used to search for the largest orthogonal spectrum to detect the true signal angle. However, there are multiple propagation paths in the indoor WLAN environment. Because the small obstacles in space will cause the signal reflection multipath effect, the path with the shortest propagation path is considered as the Line-of-Sight (LoS) path, and the other is the Non-Line-of-Sight (NLoS) path.

In a certain distance, we think the signal arrives at the receiving end with parallel waves. Therefore, the same signal will have the same phase offset Δϕ between adjacent antennas, $\Delta \phi \;=\;-\;2\pi \frac{dcos\left(\theta \right)}{\lambda }$; where θ is the true angle of each path, and *d* is the distance between two antennas. Through the signal frequency *f*, we can get the signal wavelength λ, so the wave angle θ can be easily calculated as $\theta =\arccos\left(\frac{\Delta \phi \cdot \lambda }{-2\pi d}\right)$. Assuming that the antenna array is *M* and the number of signals is *D*, the received signal *X* can be expressed as X=A·S+N, where *A* is the steering vector and *S* is the signal vector. This represents the perspective of different propagation paths of the environment. Specifically:

(3) $$A=\left[a\left(\theta_{1}\right);a\left(\theta_{2}\right);\cdots ;a\left(\theta_{D}\right)\right],a\left(\theta_{i}\right)=\left[1,e^{-j\Delta \varphi_{i}},\cdots ,e^{-j\left(M-1\right)\Delta \varphi_{i}}\right]$$

$a\left(\theta_{i}\right)$ is a column vector, and *N* is an additive Gaussian white noise with a mean of zero; the variance of $\sigma^{2}$. Therefore, the autocorrelation function of the received signal can be expressed as $R_{X}=E\left(xx^{H}\right)=AR_{ss}A^{H}+\sigma^{2}I$; where $R_{ss}$ corresponds to our signal subspace and $\sigma^{2}I$ corresponds to the noise subspace. We know that the eigenvectors corresponding to the noise eigenvalues are orthogonal to the column vectors of matrix A. The columns of A correspond to the direction of the signal. Therefore, the noise feature vector $E=\left[v_{D+1},v_{D+2},\cdots ,v_{M}\right]$. Define the spatial spectral function:

(4) $$P_{MU}\left(\theta \right)=\frac{1}{a^{H}\left(\theta \right)EE^{H}a\left(\theta \right)}$$

In Equation (4), the denominator is the inner product of the signal vector and the noise matrix. When the $a\left(\theta \right)$ is orthogonal to the E columns, the denominator is zero, and the minimum value is actually taken due to the presence of noise. $P_{MU}\left(\theta \right)$ has a spike. By traversing different angles θ, we perform the spectral search, and we can find the AoA. Three antennas can detect the angle of two propagation paths. We only need to detect the LoS path, so the receiver uses three antennas, that is M=3. Since the CSI contains 30 subcarriers, we use the covariance mean $R_{xx}$ of 30 to make it accurate.

**Figure 2:**
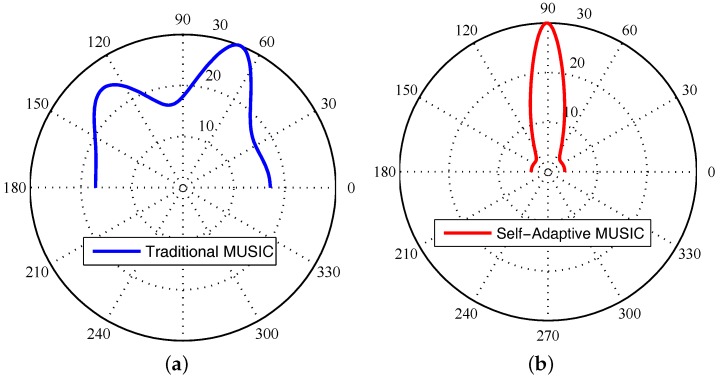
Different methods of measuring the angle of arrival. (**a**) Traditional MUSIC Algorithm; (**b**) self-Adaptive MUSIC Algorithm.

If the received signal has a component of the LoS path, the LoS path has the strongest signal strength value, then the peak of the signal’s pseudo-spectrum is the AoA of the signal on the LoS path. However, we find that the traditional MUSIC algorithm may have a big error. As we can see in Figure 2a, we test on a client that is located in $90^{\circ }$ by the traditional MUSIC algorithm. The result is an LoS angle of $69^{\circ }$, while there is an NLoS angle of $129^{\circ }$. Obviously, this is different from the $90^{\circ }$ of the tested LoS path. The reason may be that the radio frequency link connects to the RF oscillator, which will bring phase offset in CSI. However, we found that our self-adaptive MUSIC algorithm in Section 5 can accurately detect the client’s AoA when we eliminate the phase offset and eliminate the NLoS path, which is shown in Figure 2b. At this point, AoA can be accurately measured, and it can solve most, but not all of the issues in the attack model. Based on this, we propose a Sybil attack detection system.

## 4. Sybil Attack Detection System

Before the system was proposed, we found that the AoA of each client can be accurately detected when it is stationary in the experiment. However, AoA cannot be effectively measured because of multipath interference when attackers or others move in the indoor WLAN environment. Therefore, we also need to design a motion detector to resolve client motion. The amplitude information in the CSI stream is selected from the detector because it is sensitive to the motion in the environment, whereas the original CSI amplitude data are affected by noise, so it should be de-noised. As a result, we design de-noising and motion detection modules for the system.

### 4.1. Overview of the System

**Figure 3:**
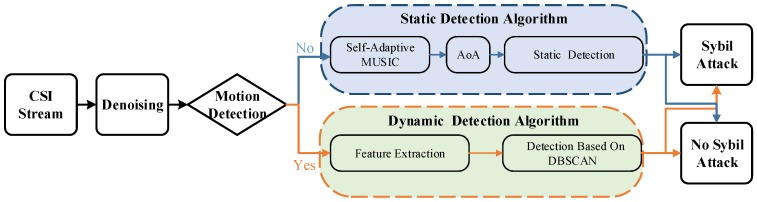
Overview of the Sybil attack detection system. CSI, Channel State Information.

At this point, we can propose a complete Sybil attack detection system. Figure 3 shows an overview of the Sybil attack detection system. According to the state of the Sybil attackers in the environment, the system can detect attacks both statically and dynamically. The system is divided into four modules:

(1) Denoising: The denoising module eliminates the noise in the CSI stream.
(2) Motion detection: The motion detection module detects the presence or absence of motion.
(3) Static detection: If all the nodes remain still, the AoA measurement is performed by the self-adaptive MUSIC algorithm. The static detection algorithm, combined with AoA, RSSI and CSI amplitude, determines whether an attack exists; because CSI amplitude and RSSI are positively related to distance.
(4) Dynamic detection: However, what if there is movement in the environment? We found that different motions correspond to different amplitude characteristics. Therefore, once there is a client movement in the environment, the dynamic detection algorithm uses the DBSCAN clustering algorithm to determine the attack after feature extraction. Only one central AP is needed to detect attack nodes by the phase and amplitude features.

**Figure 4:**
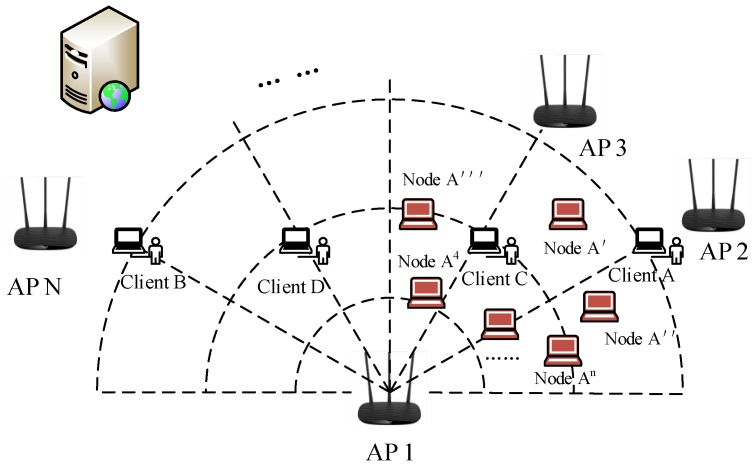
Sybil attack detection system of multiple detection APs.

When multiple APs receive CSI at the same time, they can improve the accuracy of wireless network Sybil attack detection by applying the self-adopted MUSIC algorithm and DBSCAN algorithm. As shown in Figure 4, when all the clients access the network, the central detection AP is responsible for collecting all the client data and sending the data to the server. Multiple APs can also detect if one of the centre APs is attacked by a Sybil node. Furthermore, all the attacks that we present are from indoors. If the attacker is outdoors, both the RSSI and the amplitude decrease sharply and can be easily detected. In general, experiments show that our detection system can respond to a variety of environments and achieve higher detection rates. Next, we will elaborate on each part in detail. First, the de-noising module and the motion recognition module will be explained.

### 4.2. Denoising

**Figure 5:**
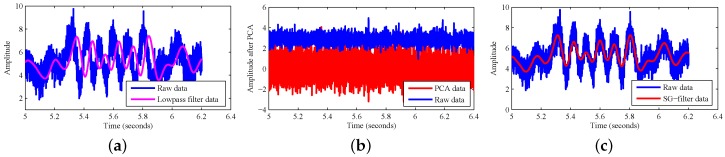
Different data processing methods. (**a**) Low pass filter; (**b**) PCA; (**c**) Savitzky–Golay (SG) filter.

The raw CSI data are subject to noise interference due to state transitions inside Tx and Rx, such as transmission power changes, transmission rate adaptation and additive DC noise of the device. These internal state transitions introduce high-amplitude pulses and burst noise in the CSI. Figure 5a shows the original CSI amplitude when the environment changes. It can be seen that the amplitude is extremely noisy. Accordingly, we should eliminate the interference noise before applying the dynamic detection algorithm.

CSI amplitude values of 30 subcarriers have different sensitivities to changes in the WLAN environment. The traditional noise elimination method uses a low pass filter, which cannot eliminate the impact of burst pulse signals in the environment. What is more, the passband of a conventional low pass filter, such as a Butterworth filter, typically requires less than 1/20 of the sampling rate to suppress the residual energy of the noise. If the cut-off frequency of the low pass filter is too high, the residual noise may distort the flow. Besides, some vibrations may contain not only low frequencies, but also high frequencies. Figure 5a shows the subcarrier signal after the low pass filter with a cut-off frequency of 80 Hz. It eliminates part of the high frequency noise, but has different effects on different carriers, leading to waveform delay. The method of Principal Component Analysis (PCA) is often used in the literature in these cases. PCA can automatically mine the correlation between CSI streams and reorganize the CSI stream to extract the change components caused by human activities. PCA can only show volatility without numerical features. Still, this method also amplifies the effects of changes in the environment. When the environment is still, its PCA value will still have much volatility as shown in Figure 5b. We use the Savitzky–Golay filter to de-noise the received data.

The Savitzky–Golay filter is a filtering method based on local polynomial least squares fitting in the time domain. The biggest feature of this filter lies in the fact that the noise can be filtered at the same time to ensure the shape of the signal and the same width. In attack detection, the waveform needs to be able to fully reflect the fluctuations of the line of sight. Therefore, we can apply the SG filter algorithm to 30 subcarriers at the same time. As shown in Figure 5c, we can see that the CSI amplitude waveform has good volatility and is smoother than the low pass filter when the SG filter is applied.

### 4.3. Motion Detection

**Figure 6:**
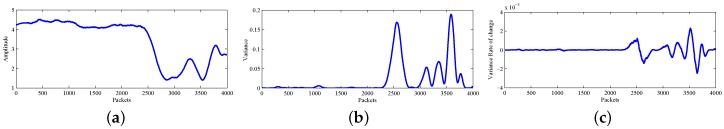
Motion detection of different packets. (**a**) Client from static to moving; (**b**) variance; (**c**) variance rate of changes.

Since the stationary node amplitude characteristics are the same, we only need to detect the time interval of the node movement. It is a challenge to determine the beginning and end of the movement. Figure 6a shows the 4000 packet amplitude data at the transmitter from standstill to walking straight with a speed of 1 m/s. The distance between transmitter and AP with Intel 5300 NIC is 4 m. Because the distance between the peaks is usually higher than 200 when there are movements, we set window size ws=200, in order to reduce the computation time and to get accurate resolution of movement. The input data can be expressed as V. When the data are less than ws, they are unavailable data.

(5) $$V=\left\{V_{1},V_{2},\cdots ,V_{i}\right\},V_{i}=\left[a_{i},a_{i+1}\cdots ,a_{i+ws-1}\right]\;,i\geq ws,a_{i}\in R$$

$a_{i}$ is the amplitude of CSI. *R* is the sequence of the entire amplitude. We calculate the variance *x* for each $V_{i}$ of the collected data. As shown in Figure 6b, it can be seen that the variance increases obviously with the fluctuation in the window. Small differences in the amplitude of the device at rest can still cause the variance to fluctuate. Therefore, we use the variance rate of variability to identify motions.

(6) $$k\left(i\right)=\frac{\Delta_{\sigma_{i}}}{\Delta_{i}}=\frac{\sigma_{i}-\sigma_{i-1}}{i-(i-1)}=\sigma_{i}-\sigma_{i-1}$$

As shown in Figure 6c, when the environment is stationary, the variance increment is almost zero, and when the node moves, the variance changes drastically. Therefore, set the threshold for *K*; when greater than *K*, then it is the beginning of the movement. The start and end points can be determined as follows:

(7) $$\begin{array}{c} Startpoint:\left[\left|k(i)>K\right|+ws/2\right]\\ Endpoint:\left[\left|k(i)\leq K\right|+ws\left(-1\right)\right] \end{array}$$

According to the attack detector, we can accurately detect whether the node is moving, so we can use the static detection algorithm to calculate the AoA by the phase feature or detect the attacks with the special amplitude feature by the dynamic detection algorithm.

## 5. Static Detection Algorithm

### 5.1. Self-Adaptive MUSIC Algorithm

In a standard WiFi network, the transmitter and receiver are not time-synchronized, so they are not synchronized to the sampling clock at the DAC and ADC. The Sampling Time Offset (STO) [12] will deviate from the arrival time and phase of the LoS path and NLoS path, which possibly cause the direct path of AoA not to be the real wave of the angle. The NLoS path may be determined as the line of sight and produces an offset. Meanwhile, when transmitting the device RF link connection to the RF oscillator, it will generate Sampling Frequency Offset (SFO). The SFO changes the sampling time offset of the same packet, which in turn leads to another additional time offset of the ToF estimate across the packet and unknown phase β.

The total time delays Δt caused by STO and SFO are very different across two packets’ transmissions between a pair of transmitter and receiver. Hence, the phase of the *i*-th subcarrier can be expressed as $\hat{\phi_{i}}=\phi_{i}+2\pi f_{i}\Delta t+\beta +N_{i}$. $\phi_{i}$ is the genuine CSI phase we are searching for, and $N_{i}$ is measurement noise. We use the classification algorithm to calculate $\phi_{i}$, and the values of Δt and β are always the same. Suppose $\phi_{i}\left(m,k\right)$ is the unwarped phase of the CSI at the *k*-th subcarrier of the *i*-th packet received at *m*-th antenna; we can obtain the optimal linear fit of the phase for the *i*-th packet as:

(8) $$\hat{\tau_{i}}=\underset{\Delta t}{\arg\min}\sum_{m,k=1}^{M,K}\left(\phi_{i}\left(m,k\right)+2\pi f_{i}\left(k-1\right)\Delta t+\beta \right)$$

The $\hat{\tau_{i}}$ is the time delay of the *i*-th packet, and we can get modified CSI phase $\phi_{i}\left(m,k\right)=\hat{\phi_{i}}\left(m,k\right)-2\pi f_{i}\left(k-1\right)\hat{\tau_{i}}$. Our method is similar to sanitizing ToF estimates in SpotFi, and this method eliminates the residual synchronization error in the CSI phase. However, this method destroys the independence of the subcarrier CSI phases so that the processed result can only be used for the digital signature, and the phase deviation of the SFO is not effectively eliminated. In order to eliminate the phase offset of the SFO and obtain a more accurate angle value, we apply the self-adaptive MUSIC algorithm to calculate the phase offset between the antennas.

We assume that the phase deviation between the antennas is $\langle \delta_{0},\delta_{1}\rangle$. Because $\langle \delta_{0},\delta_{1}\rangle$ is a hidden random variable, we cannot directly get the size of these two variables, so this algorithm uses the search method. It selects all combinations of $\langle \delta_{0},\delta_{1}\rangle$ that can be used to achieve the best combination of the reception effect as our estimated antenna deviation. We verify that we estimate the estimated antenna deviation by a number of experiments.

Because we selected better calibration results from a combination of $\langle \delta_{0},\delta_{1}\rangle$, the true value of the angle is not exactly equal to the value we measured. In addition, it is possible that not all combinations allow the peak of the pseudo spectrum to be exactly equal to the measured value. To improve the stability of the evaluation system, we need to choose a robust evaluation function to evaluate the pseudo spectrum of each combination.

- When the peak of the dummy spectrum is equal to the measured AoA value, the combination has the greatest probability of being the best calibration.
- When the peak of the dummy spectrum is close to the measured AoA value, the combination also has a larger probability of becoming the best calibration combination.

Consider the above two points; we designed our evaluation function η(ρ), where ρ is the pseudo spectrum, as follows:

- Find a normalizing constant *k* such that $\rho^{\prime }=k\rho$ is one, and set $\int k\rho \left(\theta \right)d\left(\theta \right)=1$.
- Construct a Gaussian mask $g_{\alpha }\left(\theta \right)$ with an expected value α and a variance according to the desired level of error tolerance. Set ${\bar{g}}_{\alpha }\left(\theta \right)=1-g_{\alpha }\left(\theta \right).$
- Calculate $\mu \left(\rho \right)=\frac{g_{\alpha }\left(\theta \right)\rho^{\prime }\left(\theta \right)d\theta }{{\bar{g}}_{\alpha }\left(\theta \right)\rho^{\prime }\left(\theta \right)d\theta }$.

Our algorithm estimates the best deviation for each packet and then calculates the offset of all packets. We will generate two phase deviation values $\langle \delta_{0},\delta_{1}\rangle$ in the multiple clustering algorithm and select the most frequent phase deviation combination as our final estimate. Calibrate Antenna 2 and Antenna 3 while applying the calibrated CFR to the traditional MUSIC algorithm. This inherent deviation occurs when the device is started and does not change when the device is running, but the phase deviation is reset when the device is restarted. We apply this algorithm to the spatial smoothing MUSIC algorithm to automatically correct each phase offset at the start of the device.

At this point, we eliminate the time offset in the different CSI packets generated by the STO, make the direct path more accurate and evaluate the optimal phase offset by the self-calibration method, which is generated by the SFO. In the fourth quarter, a large number of experiments shows that for different angles of the AP, the self-calibration algorithm can effectively detect its position.

What we want is only the direct path. Therefore, we adopt spatial smoothing to get the direct path accurately. Though our witnessing AP only has a three-antenna array, it can detect two AoA in the indoor environment. Therefore, we group our antennas into two groups {antenna1,2;antenna2,3} as the input signal to detect the direct path. In detail, spatial smoothing generating signals $x_{1}$,$x_{2}$,$x_{3}$ would output two signals ${\dot{x}}_{1}$, ${\dot{x}}_{2}$ where ${\dot{x}}_{i}=\frac{1}{2}\left({\dot{x}}_{1}+{\dot{x}}_{2}\right),i=1,2$. At last, we implemented two algorithms using only a three-antenna service AP. Testing the same situation as the traditional algorithm, that is the $90^{\circ }$ AP on the LoS, our self-adaptive algorithm can accurately detect the LoS, and the detection error is $1^{\circ }$, which shown in Figure 2b. At the same time, the impact of NLoS is eliminated. Compared to Phaser [14] and ArrayTrack [44], we use fewer antenna arrays and achieve the total average error of $6.3^{\circ }$; accurately enhancing $5.6^{\circ }$ compared to traditional algorithms. Similarly, compared with the SpotFi [10] with a certain complexity, we do not need the clustering algorithm to calculate the AP position and ToF estimation.

### 5.2. Static Sybil Attack Detection

**Figure 7:**
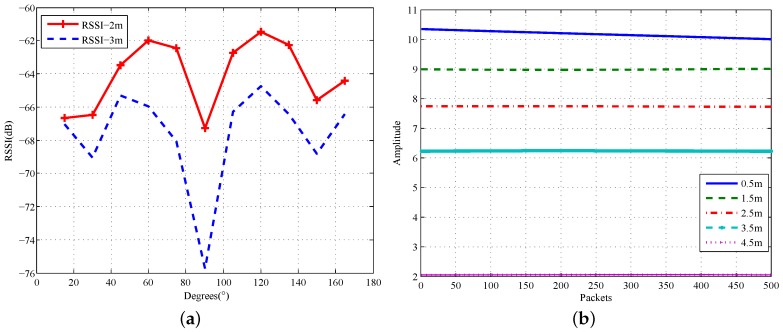
Amplitude and RSSI of different distances. (**a**) RSSI of different distances; (**b**) amplitude of different distances.

The self-adaptive MUSIC algorithm can accurately calculate the AoA of each client when they are in a static state. However, different clients cannot be distinguished only by their AoAs if they are in the same angle, but not in the same position. Fortunately, CSI packets contain not only distance-related RSSI of different channels, but also stable amplitude information at the physical layer. Suppose node *i* receives a radio signal from Node 0, then the RSSI is $R_{i}=\frac{P_{0}K}{\alpha \cdot d_{i}}$ where $P_{0}$ represents transmitter power, $R_{i}$ is RSSI and *K* is the constant of the impulse response of the Rayleigh channel model. $d_{i}$ is the Euclidean distance, and α is the distance-power gradient. Hence, at the same transmit power, the RSSI is inversely proportional to the distance *d*. As we can prove in Figure 7a, we compare different angles’ total RSSI of the 30 subcarriers in the same radius of 2 m with that in radius of 3 m. We can see clearly the gap between the RSSI of different degrees. The average difference is 1.33 dB. In addition to the $15^{\circ }$ gap being relatively small, the other degrees of RSSI differences are more than 2.6 dB. Therefore, we can distinguish between nodes in different angles at different locations effectively.

Some unstable RSSIs may result in completely different values for different time periods. However, on the other hand, CSI energy does not change with time; the CSI energy decreases with the increase of distance, that is the amplitude of the client decays with distance. Figure 7b shows the average amplitude of Subcarrier 1 with a time window of 0.2 s. It can be seen that the magnitude of CSI is approximately inversely proportional to the distance, as the distance increases from 0.5–4 m by 1-m steps. Moreover, the energy at the receiving antenna decays drastically at a location of 4.5 m, which is caused by the increase of the NLoS path reflection. Experiments show that the CSI amplitude values decrease by more than 1.4 per meter. Therefore, we set this value as the amplitude threshold for different positions. At this point, we can accurately distinguish the client in different locations and degrees. It also avoids the instability of RSSI by combining CSI amplitude information. For a further explanation, we will describe in detail the specific Sybil attack model in Figure 7b to see if our algorithm can effectively detect the attacker.

For the case of Figure 1a, Client A falsifies the identity of Client B as node $B^{\prime }$ and sends a request message to steal the returned data sent from AP 1. Since AP 1 receives the CSI phase information sent by node $B^{\prime }$, AP 1 can locate the client for the actual sending data by combining our improved MUSIC algorithm. That is, Client A is located in the direction of $30^{\circ }$. We can be sure that client B’should be identified as a Sybil node. Figure 1b and Figure 1a are similar, the only difference is that Client A and Client D are at the same angle, but have different radii. Thus, while our MUSIC algorithm can determine that $D^{\prime }$ and D have the same angle, they have different RSSI values. Node $D^{\prime }$ can also be determined as a Sybil node.

In the attack model of Figure 1c, when A falsifies a large number of Sybil nodes, AP 1 does not care about the contents of the packet sent by the virtual node and only determines the angle of the client based on the CSI by our algorithm. If the number of nodes does not affect Client B’s, Client C’s and Client D’s access to the network, we can determine that there are only four angles in the network sending the data, and they are at [$30^{\circ }$, $150^{\circ }$, $60^{\circ }$, $120^{\circ }$]. When Client B, Client C and Client D suffer from DoS attacks due to a large number of nodes, we can only detect that there is only one node in the network where the data are sent, and it is at $30^{\circ }$, so we can determine that the remaining nodes are Sybil nodes, which forged Client A.

However, the constant movement of attackers interferes with the channel phase information so that Sybil nodes’ AoA calculated by the self-adaptive MUSIC algorithm will change within a large area. Therefore, it cannot reflect the real AoA of the Sybil client movement. Moreover, when there is more than one client movement in the environment, the detection accuracy of the static detection algorithm will be reduced. Therefore, we propose a dynamic detection algorithm to solve the situation where there are movements of clients in the WLAN environment.

## 6. Dynamic Detection Algorithm

As we mentioned earlier, each client will not always remain stationary in an indoor wireless environment. Although our static detection algorithms are effective against Sybil attack in a static position, attackers may constantly move their position to change their channel characteristics, adding extra noise to the phase values. According to the literature [45], the CSI amplitude can sense the movement or gesture in the environment. Therefore, we can analyse the attack behaviour of malicious movement according to different fluctuation characteristics provided by different movement characteristics.

**Figure 8:**
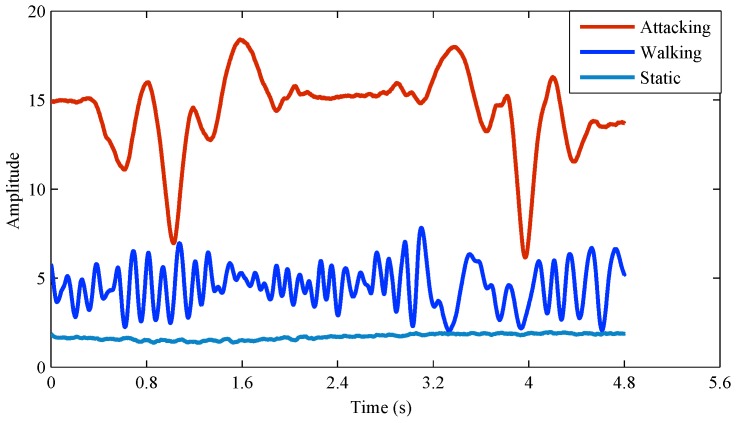
Different activities in the environment.

The CSI amplitude has a stable characteristic when there is no movement of the environment. Its value basically remained stable. However, the amplitude of CSI will exhibit periodic fluctuations as the device moves. Figure 8 shows that the amplitude of the physical layer varies differently for different activities. Because the waveform moves in the person or device, the reflection path will be produced in different ways. As a result, the peaks and valleys will add up or down in the same direction over a period of time. Both clients and attackers may exhibit some fluctuations in the real environment. In addition, each client moves at a different frequency, which will only respond to a particular change in amplitude waveform. Attackers will present extremely similar waveforms. As Sybil attacks fake nodes from the same physical node, the forged packet information and forged packet structure will not affect CSI amplitude changes.

### 6.1. Feature Extraction

We know that attackers’ movements have a specific behavioural trait. In fact, they launched such acts in order to avoid the static detection algorithm. It is a feature that attackers move their positions slightly to change the CSI phases. This is different from the actual client’s movements. The motion detection algorithm can detect the starting and ending moments of any client’s motion. What we need to do is to collect the datasets at each node attack and in the absence of an attack, according to different node characteristics.

Let $X=\left\{x_{1},x_{2},x_{3},\cdots ,x_{j}\right\},j=200$. $x_{j}$ is the different client amplitude data within 2 s from start to end when there is no Sybil attack. $x_{i}$ is zero if there is no movement or no client access. We simulated the attack so that one of the nodes became a Sybil client, forging 50 attack nodes. Repeat the same action and the same kind of action in 2 s to get the dataset $Y=\left\{y_{1},y_{2},y_{3},\cdots ,y_{j}\right\},j=200$. We calculate the eight statistical features in each of $x_{i}$ and $y_{i}$ from *X* and *Y*, which are the Maximum (Max), Minimum (Min), Variance (Var), Range, number of times the Signal Crosses the Mean value (MCR), the Standard deviation (Std), the Area under the signal curve (Area) and the total Number of Peaks and Valleys (NPV). We assume that each node has the same probability of moving. Let $z=\left|x_{j}-x_{j+1}\right|$, then P(z) can be expressed as:

(9) $$P\left(z\right)=\frac{count(z>k)}{count(g=1)}\cdot \frac{g}{200},g=\left\{\begin{array}{c} g=1,\;if\;x\neq 0.\\ g=0,\;if\;x=0. \end{array}\right.$$

The probability of other cases is 1−P(z), so the entropy H(z) of different features is expressed as:

(10) $$H\left(z\right)=-\left[p\left(z\right)logp\left(z\right)+\left(1-p\left(z\right)\right)log\left(1-p\left(z\right)\right)\right]$$

The entropy of x and y is equally valid. Therefore, the information gain after launching the Sybil attack is $IG\left(z_{y}|z_{x}\right)=H\left(z_{y}\right)-H\left(z_{y}|z_{x}\right)$. IGfor normal situations can be calculated by $IG\left(z_{x+1}|z_{x}\right)=H\left(z_{x+1}\right)-H\left(z_{x+1}|z_{x}\right)$, and $z_{x+1}$ is the data increment that changes only one normal client to one attack node. At the same time, we also perform PCA processing on the data of 30 subcarriers after normalizing the data and select the first major feature and calculate its eight features. The results are shown in Table 1.

Because each node has the same probability of moving, the Sybil attack will generate a large number of attack nodes. Therefore, the probability of Sybil nodes occurring is higher than that of other nodes, and the information entropy increases as the attack nodes increase. As for the information gain, attack nodes faked by Sybil attacks belong to the same physical layer nodes. Therefore, once the attack exists, IG will remain almost unchanged. On the other hand, the dimensions of the 30 subcarriers are too redundant, and most of the motion-sensitive carriers are mostly fixed. The PCA-based method does not change the amplitude characteristics of the physical layer in the environment because of the Sybil attack. Therefore, in summary, it can be seen from Table 1 that the range and the total value of packs and valleys are consistent with the Sybil attack characteristic, so the two eigenvalues are selected as the input features. In fact, this is because attacks are less frequent, making the number of NPV per attack nodes be far less than the normal activity node, and the range is relatively large.

### 6.2. Density-Based Spatial Clustering of Applications with Noise

When the Sybil attack occurs, a large number of attack node amplitude samples is closely distributed. Moreover, the motion characteristics are different from those of normal nodes. However, we are not sure of the number of actual nodes in the environment, and there are nodes that are accessed and offline at any time. According to [45], in the frequency domain, the frequency is about 35–40 Hz, and the wavelength is 5.15 cm when a person equipped with any WiFi device in WLAN is walking. Its frequency range is 40–80 Hz when he or she falls. The frequency is 15–30 Hz when a person carries the WiFi device in WLAN standing up and sitting down. We define these moved WiFi devices for mobile clients. However, the attacks will be repeated periodically and mostly below the normal user behaviour frequency. The frequency is 2–5 Hz. Combined with experiment and feature selection results, we find that the time domain can provide richer motion characteristics when the amplitude is effectively filtered.

Usually, the attacker is at a low frequency. In fact, if the attacker moves more aggressively or if the mobile device user happens to perform a low-frequency movement, the frequency domain-based attack identification will be confused. However, in fact, there are many forged nodes in the Sybil attack. The main feature is that a large number of those nodes has the same physical layer characteristics. Therefore, based on this feature, we can calculate the attacker cluster of high density and effectively distinguish attackers and normal clients. The features NPV and range are more accurate than frequency and can display distinctly different clusters. However, they do not always stabilize for a particular value. Therefore, not every cluster is a spherical data distribution. We use Density-Based Spatial Clustering of Applications with Noise (DBSCAN) to cluster the Sybil attack nodes. DBSCAN is able to cluster the same movements in closely-spaced density into one cluster and does not need to enter the category number. What is more, it can find any shape of the cluster and noise points while clustering.

In detail, the core of DBSCAN is to find the largest set of samples with the highest density connected to each other by density-reachable relations. We cycle through all the packages within multiple time windows. The average of range and NPV is clustered in each time window. Because we use the 5-GHz wireless band, the sampling frequency can be set as 2500 packets/s, which ensures that each motion feature can be richly collected. Features will not change significantly within 1 s; therefore, the rectangular window length ws=2500. The motion features per second of *m* clients are $r_{m+n\cdot ws}$; $r_{m}$ is client *m*’s features of the first second, and *n* is the data acquisition time in seconds. If the data length is less than the length of the window, it is considered as unavailable data. The input is:

(11) $$W=\left\{r_{1},r_{1+ws}\ldots r_{1+n\cdot ws},r_{2},r_{2+ws},\ldots ,r_{2+n\cdot ws},\ldots ,r_{m+n\cdot ws}\right\},r\in R^{2}$$

The output is the cluster number *C*. The ε-neighbourhood is the Euclidean distance between every two samples; the threshold is ε; and MinPts is the minimum number of pairs in the neighbourhood. If it is greater than this number, the point is the core point. Then, the same cluster can be reached by density-reachable and density-direct means. The algorithm is shown in detail in Algorithm 1.
**Algorithm 1:** Density-based spatial clustering of applications with noise.
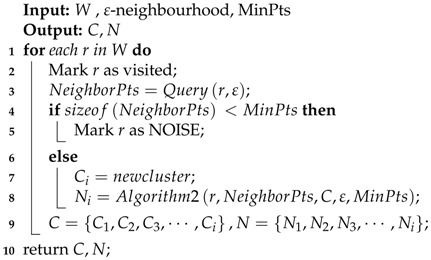


The function query is to query the number of points in the neighbourhood of *r*. Algorithm 1 calculates the final result of DBSCAN, which is the clustering cluster and the density-reachable points within each cluster. Algorithm 2 belongs to Algorithm 1, which is the core of DBSCAN. Algorithm 2 calculates all the density-reachable points of all the neighbourhood core points. It is assumed that the AP collects the amplitude characteristics of M clients by receiving CSI. If these movement nodes have different MAC addresses, there is no behaviour such that Sybil nodes copy the real node’s MAC address, but it can not prove that there is no Sybil attack. An attacker can randomly forge large numbers of MAC addresses that do not exist in the network. We need further validation, therefore, if the number of clusters calculated by Algorithm 1 is less than M, then the clusters with higher density can be regarded as forged Sybil nodes. If there is a different client with the same MAC address, we can determine that there is a Sybil attack on the network. Sybil nodes in W can also be returned by Algorithm 1.

The only difference between the common motion feature and the attack motion feature is that in the same time window, the forged attack nodes are all from the same attacker, so the density within the attack cluster is much higher than that of the common motion nodes.
**Algorithm 2:** Calculate all density-reachable points of core points.
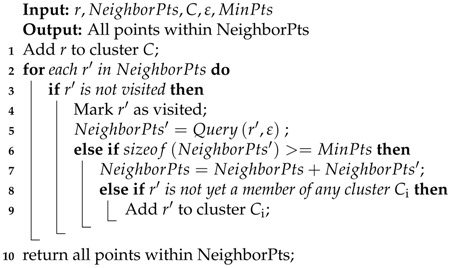


## 7. Experimental Evaluation

Our experimental environment is a 6.35 m × 8.5 m meeting room. Receivers and transmitters are mini-PCs equipped with Intel 5300 WiFi NICs. They all contain three antennas. We use one antenna at the transmitter and three antennas at the receiver. We only employed one receiver. We installed CSI tools [46] on these mini-PCs, which can receive the CSI of 30 subcarriers. In order to eliminate the interference in the environment, while further reducing the impact of phase offsets, we use the monitor mode and 5.32-GHz band. Our system can not only work in the 5-GHz band, but also in the 2.4-GHz band. We only need the centre AP equipment with WiFi NICs, which can receive CSI signals. Regardless of whether sending any type of WiFi device and any type of packet, we can receive the CSI when it accesses the wireless network and sends it to the transmitter. We use the same experimental environment for both dynamic and static detection algorithms.

We conducted a total of five sets of experiments on Sybil attack detection from static and dynamic scenarios, respectively. Three of them are static attack detection experiments, which are respectively the AoA accuracy comparison of different algorithms, the AoA error estimation of different packets and access points and the detection accuracy of different types of Sybil nodes in a static scenario. Dynamic attack detection experiments include two sets, that is the accuracy of the DBSCAN clustering algorithm and the detection accuracy of Sybil nodes.

In detail, we test an AP and mobile phone, which support the 802.11a/c protocol, as the AoA of the transmitter and use the mini-PC with the Intel 5300 network card as the receiver. In the static detection algorithm, the AoA value is set according to the experimental threshold, and the RSSI and amplitude detection threshold are combined to verify the efficiency of our Sybil node detection algorithm. We then use MATLAB simulation to increase the number of Sybil attacks compared to the RSSI attack detection methods. When applying dynamic detection algorithms, we let the client implement a variety of motions and verify the accuracy of the DBSCAN algorithm separately. Then, we increase the number of movement attack nodes and verify that the algorithm is still able to effectively detect when there are many kinds of movement patterns in the environment.

### 7.1. Angle of Arrival Estimation Accuracy of Different Clients

**Figure 9:**
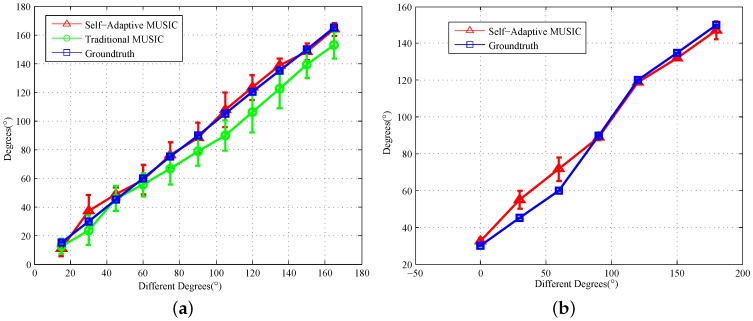
AoA Error bar of different clients. (**a**) Error bar of access points; (**b**) error bar of mobile devices.

In this part of the experiment, we tested AoA’s accuracy on 5G band clients, including APs and mobile phones. Let the transmitter be at 11 degrees, which ranges from $15^{\circ }$–$165^{\circ }$ by steps of $15^{\circ }$. The distance between the transmitter and the receiver is 3 m. After collecting the CSI data, we changed the distance to 2 m and carried out the same experiment. Through our improved MUSIC algorithm, we can determine the inherent phase offsets $\langle 8^{\circ },20^{\circ }\rangle$ of Antennas 2 and 3. Figure 9a shows the error bar of the traditional MUSIC algorithm and our algorithm in different angles’ measured data, which randomly selected 300 packets. We can see that the average of our algorithm is almost the same as the true angle with the average error being $6.3^{\circ }$, but the average error of the traditional operator is $11.9^{\circ }$. For mobile phone testing, we changed the experimental environment to the office, filled with desks and computers. The size is about 64 $m_{}^{2}$. Then, we put the phone and the antennas at the same level of height and adjusted the distance between the receiving antennas to 6 cm to prevent interference between the receiving antennas. Due to the many obstacles in the environment, we are making sure that the external environment has not changed. On the semicircle with a distance of 1 m, we changed the position of the mobile phone [$30^{\circ }$, $45^{\circ }$, $60^{\circ }$, $90^{\circ }$, $120^{\circ }$, $135^{\circ }$, $150^{\circ }$] as the measurement angles, respectively. Figure 9b shows that we can also effectively detect the angle of the phone in the wireless network with an accuracy of $7.2^{\circ }$. The average RSSI difference in different locations is 1.6 dB.

**Figure 10:**
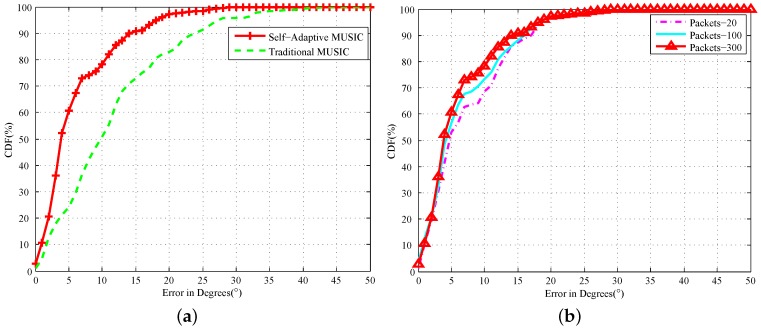
AoA Estimation error. (**a**) CDF of access points; (**b**) CDF of access points of different packets.

Figure 10a shows the AOA’s CDF estimate error with all APs, and we can see that 80% of the AP’s detection error in 300 packets does not exceed $10^{\circ }$, whereas the traditional algorithm is only 50%. We simulate the number of different packages as shown in Figure 10b, and the experiment shows that our algorithm can effectively improve the positioning accuracy with the CSI packets’ increase. The average RSSI of 8000 packets, shows that the difference between 2 m and 3 m is 1.3 dB. The CSI amplitude values decrease by more than 1.4 per meter.

### 7.2. Detection of Sybil Nodes in the Static Scenario

In the same experimental environment, 20 sets of different nodes are randomly arranged at positions of 2, 3, 4 and 5 m and the angles of [$30^{\circ }$, $60^{\circ }$, $90^{\circ }$, $120^{\circ }$] for Sybil attack detection. According to our Sybil attack model for multiple experiments, we set $6.3^{\circ }$ degrees as the AoA threshold, 1.3 dB as the RSSI threshold and 1.4 as the amplitude threshold. Because we only use one AP as a detection node, the RSSI-based Sybil attack detection efficiency is 76.5%; whereas our detection system based on CSI has a detection rate of 100%. The experimental results have a relatively high accuracy, due to the large differences between the angles of different locations, and the test dataset is relatively small. However, this proves that the accuracy of our measurement is much higher than RSSI. For further research, we assume that we can detect the nodes in the error range of AoA, RSSI and CSI amplitude based on the above ideal environment.

**Figure 11:**
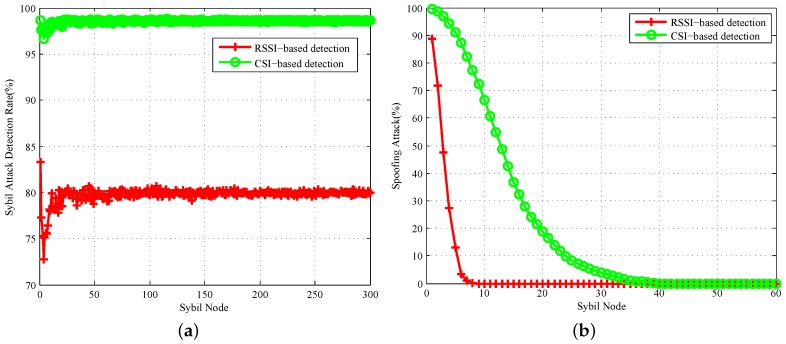
Different attacks’ Sybil node detection rate. (**a**) Sybil attacks’ Sybil node detection rate; (**b**) spoofing attacks’ Sybil node detection rate.

In order to further explore, we use MATLAB to randomly generate a large number of nodes with AoA, RSSI and CSI amplitude. We set 500 WiFi clients randomly and simulate Sybil attacks 200 times. We can detect whether the Sybil attacks occur accurately or not as the Sybil nodes increase. Figure 11a shows the detection rate of the virtual Sybil nodes generated by Sybil clients. We can see that our algorithm can achieve an average detection efficiency of 98.5% for the Sybil nodes, and the RSSI detection method can only achieve 79.8%. We define that the identities of multiple fake Sybil nodes in spoofing attack are all the real clients existing in the network. With the number of spoofing clients’ Sybil nodes increasing, Figure 11b shows the probability that each node is detected by a spoofing attack. When the spoofing nodes are four, our detection efficiency is 94.2%. With the increase in spoofing at the same time, our accuracy is gradually declining. When a client is virtual out of 40 Sybil nodes, we cannot accurately distinguish every node generated by clients of spoofing attacks. However, we can still know that some nodes are being attacked. Spoofing attack detection needs to detect all fake nodes, which is a factor that limits the detection rate. Therefore, as long as there is a node that has not been accurately detected, we think that the entire test is a failure.

### 7.3. Accuracy of DBSCAN Clustering Algorithm

Each client connected to the central AP is a normal client and does not initiate a Sybil attack. Clients perform 12 different types of periodic movements or rest at random locations for 10 s, respectively, when connecting the central AP, including walking fast and slowly in a small area, sitting down fast and slowly, jumping, running in place, boxing, fighting, turning quickly and slowly, crouch and moving slightly (simulating an attacker’s behaviour). So far, we have collected a validation dataset through which we can verify the clustering characteristics and attack detection accuracy of the dynamic detection algorithm when there are multiple motions in the environment.

**Figure 12:**
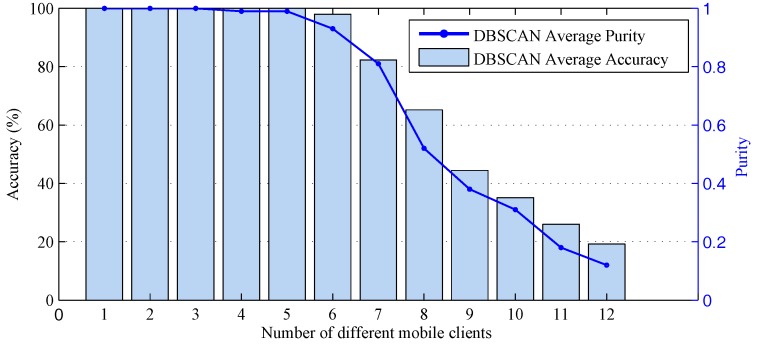
Different mobile clients in the environment.

Figure 12 shows the DBSCAN clustering algorithm’s accuracy when there are different numbers of mobile clients. The algorithm can distinguish the different categories with an accuracy of over 82.7% when there are seven and fewer movements. However, when there are more than seven types of client movements, the accuracy of the clustering and the purity of the clusters are drastically reduced. At the same time, there are no more than seven kinds of movements in most cases of WLAN. The most obvious features of those movements are walking fast and slow, jumping, turning quickly and slowly, moving slightly and sitting down slowly. The purity of these clusters is higher than 0.81, which can be used as a basis for the dynamic attack detection of client actions. The limitation is that these clients cannot move too far away from the original position, and their motions need to be consistent. Therefore, the DBSCAN algorithm can effectively distinguish the usual movement in the indoor WLAN environment. At the same time, when the number of motions increases by more than seven, some nodes will gather, reducing the detection accuracy due to the similar amplitude characteristics of other clients.

### 7.4. Detection of Sybil Nodes in the Dynamic Scenario

When simulating Sybil attacks, we allow attack clients to access and leave APs within a certain number of times. Attack clients forge multiple nodes and periodically move in their location. Similarly, we make other clients move randomly, and the attacker also makes periodic dynamic attacks at a specific time. We collect a total of 12 datasets each for 10 s. We calculate the Euclidean distance between each feature point and all other points to get the ε-distance set and then sort them in ascending order. We use a scatter plot to show the ε-distance trend and finally determine the value of the radius MinPts based on this. Finally, we get the optimal clustering parameters (0.5, 30). At this point, the clustering effect of attacks is most obvious, and according to the dataset, we can see that the clustering clusters of motion features of a single client will not exceed 120. Then, we set the value as a threshold. When the cluster is above the threshold, it is determined that the node in the cluster is the attacking node. The number of types of motion in each dataset is fixed.

**Figure 13:**
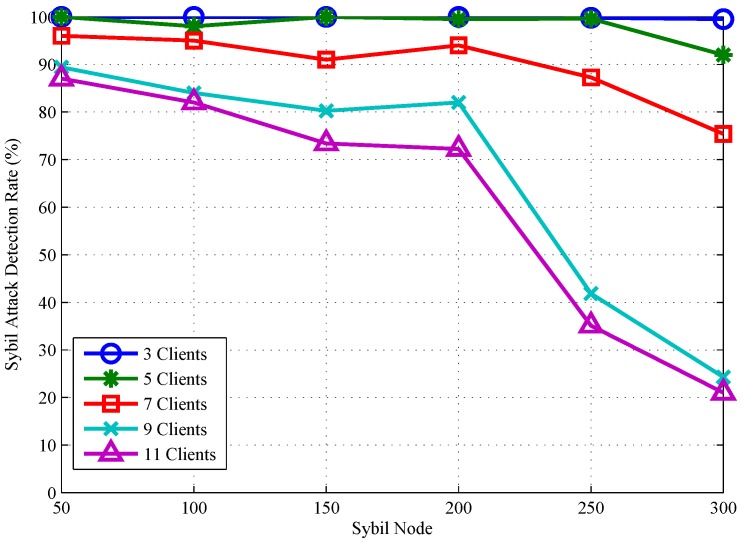
Dynamic Sybil attack detection rates for different mobile clients.

Therefore, we also increase the variety of clients in the environment to move in different ways and increase the number of fake attack nodes, as shown in Figure 13. Experiments show that the more attack nodes, the greater the density of their clusters, so the accuracy of attack detection increases with the number of attack nodes. Especially when there are five clients moving in the environment and fewer than 250 forged nodes, the overall average detection efficiency is 99.5%. This is because attack nodes forged by the actual attacker fluctuate within a small area and cannot be connected to other types of clusters. The attack detection rate remains high despite the increase in attack nodes. As the number of mobile clients increased to 9, 11and the number of forged attack nodes was less than 200, the algorithm could not cluster all the nodes accurately, but still could detect a large number of simulated nodes with the detection rate still above 75%. However, with the increase of simulated attack nodes, some large density clusters will swallow many small clusters. Algorithms are easier to cluster into one. This misjudgement makes the detection accuracy drop acutely.

In conclusion, the dynamic detection algorithm can maintain a high detection accuracy for less than 250 attacker forged nodes and meanwhile can achieve an average detection accuracy of 99.5% when there are less than five kinds of motion in the environment.

## 8. Conclusions

Aiming at privacy leakage, identity theft and wireless DoS attacks that Sybil attacks may bring, this paper proposes a detection system that can detect Sybil attacks based on fine-grained physical layer channel state information. Moreover, in this system, we combine the self-adaptive MUSIC algorithm with the CSI amplitude for static attacks and propose a detailed feature extraction method with DBSCAN to detect dynamic attacks. Experiments show that the system can achieve a high detection rate for both static attacks and dynamic attacks. According to our experimental results, the average detection rate of the static algorithm is 98.5%, and the average detection rate of the dynamic algorithm is 99.5% as long as no more than five clients are moving. However, the dynamic detection algorithm is greatly disturbed by the environment. When people without WiFi devices are moving in WLAN, this will affect the acquisition of data and the detection accuracy. At present, we cannot effectively distinguish the movement without the WiFi device, which will be carried out in our future work. 

## Figures and Tables

**Table 1:** Effect of the attack on various feature selection schemes. MCR, number of times the Signal Crosses the Mean value; NPV, the total Number of Peaks and Valleys.

**Normal State**									
	**Features**	**Min**	**Max**	**Var**	**Range**	**MCR**	**Std**	**Area**	**NPV**
**Schemes**									
I		0.040	0.039	0.059	0.082	0.129	0.186	0.246	0.048
IG		0.019	0.021	0.042	0.045	0.085	0.106	0.190	0.039
PCA		3.350	−1.530	1.712	4.880	3	1.311	1.215	4
**Attack State**									
I		0.051	0.038	0.085	0.143	0.135	0.193	0.208	0.151
IG		0.045	0.029	0.027	0.054	0.126	0.087	0.115	0.040
PCA		3.490	−1.340	2.132	4.830	4	1.462	0.700	4
